# Formation of a Pt-Ni Catalyst in the Structure of a Silicon Micro-Fuel Cell

**DOI:** 10.3390/molecules31030499

**Published:** 2026-01-31

**Authors:** Vitaliy V. Starkov, Ekaterina A. Gosteva, Alexey Kartsev, Svetlana V. Agasieva, Sorokin I. Dmitry

**Affiliations:** 1 Institute of Microelectronics Technology and High-Purity Materials, Russian Academy of Sciences, 6 Academician Ossipyan Str., Chernogolovka, Moscow 142432, Russia; 2 Peoples’ Friendship University of Russia—RUDN University, 6 Miklukho-Maklaya Str., Moscow 117198, Russia; 3 National University of Science and Technology MISiS, 4 Leninsky Prospekt, Moscow 119049, Russia

**Keywords:** “core-shell” structure, composite catalysts, proton exchange membrane, micro-fuel cells, gradient porous silicon

## Abstract

This paper demonstrates the results of constructive technological research on the development of a catalyst with a Ni/PSi@Pt structure. This catalyst eliminates the use of gold in the structure of μ-FC electrodes. This work uses the main technological solutions for the formation of a gold-containing “core–shell” structure on the inner surface of pores. Comparative data on the results of assessing the durability of porous silicon electrodes with both Pt catalysts and composite catalysts of the Pt/In_2_O_3_, Pt/SnO_2_, Pt/Au and Pt/Ni types are also presented.

## 1. Introduction

The energy capacity of commonly used consumer electronic power supplies today is typically between 30 and 80 watt-hours per kilogram for nickel–cadmium and nickel–metal hydride batteries and between 100 and 250 watt-hours per kilogram for lithium-ion and lithium-polymer batteries. A proton exchange membrane fuel cell (PEM FC) can produce 800–1300 watt-hours of energy per kilogram. Thus, the energy intensity of low-temperature fuel cells exceeds the capacity of known batteries by 5–10 times. This is one of the key considerations that explains the increased scientific efforts to create miniature power supplies based on PEM-FC. Such power supplies with a power of up to 25 W belong to micro-fuel cells (μ-FC). Progress in the creation of industrial technology μ-FC is associated with the development of a design and technological option based on silicon and, in particular, porous silicon, as well as technologies for carbon nanotubes and graphene, nanocomposite membranes, and electrocatalysts [1,2,3,4,5,6,7,8,9,10]. One of the most miniature μ-FCA designs is described in [1]. The authors developed a unique 9 μL power supply (3 × 3 × 1 mm^3^). The source also includes an autonomous hydrogen source using the reaction between the metal hydride LiAlH4 and water. The resulting hydrogen passes from the reactor through a nanoporous silicon membrane and reaches the μ-FC membrane electrode unit. For 32 h, this source is relatively stable in providing 254 W power to the load. The disadvantage noted by the authors is the low duration of the source’s work. A significant impact on the durability of μ-FC is exerted by drop-in platinum catalyst activity after some time of effective operation. In this structure, the platinum catalyst interacts directly with the porous layers of μ-FC silicon electrodes. To ensure the required durability, it was proposed to place the catalyst on the surface of porous silicon (PSi) after pretreatment with various primers. Analysis of the catalytic activity of the Pt catalyst after various treatments of the pore surface was conducted in [2]. A significant increase in the duration of effective functioning of the Pt catalyst after pretreatment of the pore surface using a gold-containing primer was demonstrated. A process was proposed that makes it possible to synthesize a nanomaterial with a core–shell structure (Au/Si@Pt) on the inner surface of deep pores (200–350 μm). In the structure of pores with a diameter of 0.8–3 μm, a dispersed structure of gold/silicon nanoislands is formed, forming a “core”. The structure’s “shell” is made up of a platinum catalytic layer. High resistance to harsh operating conditions was demonstrated by the Au/PSi@Pt catalyst in a fuel cell’s half-cell structure. There was almost no drop in the open circuit voltage (U_0_) value over the course of the experiment, which lasted more than 700 h.

This paper demonstrates the results of constructive technological studies on the replacement of a catalyst with an Au/PSi@Pt structure with Ni/PSi@Pt. This catalyst eliminates the use of gold in the structure of μ-FC electrodes. This work used the main technological solutions for the formation of a gold-containing “core–shell” structure on the inner surface of pores. Data on the durability tests of electrodes with Ni/PSi@Pt catalyst are also presented.

## 2. Experimental

### 2.1. Research Methods

The structure of catalysts on silicon electrodes was studied using optical, electron, and atomic force microscopy, as well as X-ray diffractometry and energy-dispersive analysis. The elemental composition of the catalysts was also determined via mass spectrometry with inductively coupled plasma (X-7 ICP-MS, Thermo Elemental, Waltham, MA, USA installation).

For a comparative assessment of the electrocatalytic activity of platinum on silicon electrodes, the open-circuit voltage (U_o_) in the FC half-cell was measured in the hydrogen-air mode at the cathode electrode. A (0.5–1) M H_2_SO_4_ solution is used as an electrolyte; hydrogen is supplied to a platinum reference electrode.

### 2.2. Silicon Electrodes

The experiments used silicon electrodes in the form of polished silicon wafers and porous membranes based on two-layer gradient porous structures (GPSi-2 structures [3]) and porous structures with variable porosity (GPSi-var structures [4]). The morphology of the pores of the GPSi structures is characterized by a significantly developed surface and a high aspect ratio. This kind of phenomena determines the increased interest of FC developers in such a structure of electrodes.

The proposed GPSi structures as μ-FC electrodes provide the necessary mechanical strength of the membranes during the subsequent assembly of the μ-FC working structure [3,4].

After the formation of the porous structure and the removal of the unused silicon layer, the porous membranes were chemically cleaned. Subsequent processing is associated with the formation of a catalytic Ni/PSi@Pt layer in the porous structure of silicon membranes.

### 2.3. Formation of Ni Primer on the Porous Surface of Silicon

**Figure 1:**
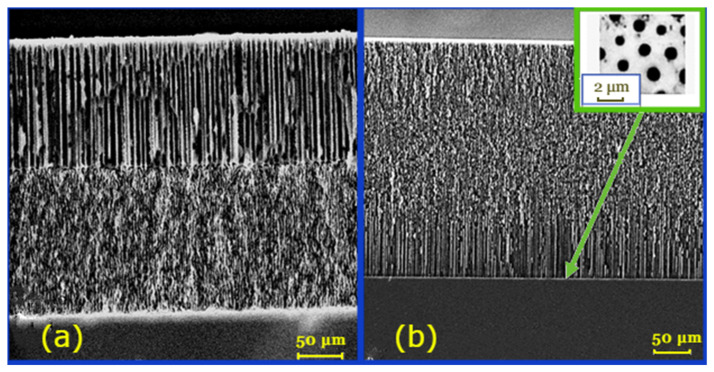
SEM images of the transverse cleavage of GPSi structures. (**a**) GPSi-2 structures: the thickness of the upper layer is 130 μm, the pores have columnar morphology, and the average pore diameter is 5 μm. The second layer has a spongy pore morphology, is 150 μm thick, and is 1.5–2 μm in diameter. (**b**) GPSi-var structures: the inset shows an optical image characterizing the topological arrangement of columnar pores at the bottom of the porous membrane after the removal of unsealed silicon.

Enhancing the catalyst’s efficiency significantly improves the fuel cell’s overall performance. Platinum-based catalysts are employed in hydrogen-air and methanol fuel cells for the electroreduction process of oxygen. The application of Ni as a carrier for catalytic Pt in commonly utilized carbon fuel cells has been extensively examined in the scientific literature [11,12,13,14]. The issue of Ni sublayer development for platinum catalysts within the μ-FC silicon electrode structure is often overlooked. Nickel is intriguing as a construction material due to its classification as a transition metal. It possesses a comparatively diminutive atomic radius, (Ni—0.124 nm). Consequently, when deposited on the inner surfaces of the pores (e.g., GPSi-2 Figure 1a), their shape and the preferential orientation of the outer layer perpendicular to the (100) surface are maintained. Moreover, nickel and its oxide possess catalytic capabilities, are economically advantageous, and are more abundantly available in nature than noble metals. Nickel was deposited on the pore walls of silicon through impregnation of the porous layer. This approach is straightforward and economical, yet simultaneously very productive and successful for the functionalization of porous medium [15]. During impregnation, the samples were submerged in an alcoholic solution of nickel sulfate (NiSO_4_⋅7H_2_O) and maintained for the duration required for nickel to precipitate over the entirety of the pores. The percentage of isopropyl alcohol was selected to ensure that the electrolyte solution thoroughly wetted the surface of the porous silicon. Following deposition, the samples were extracted from the solution and air-dried.

**Figure 2:**
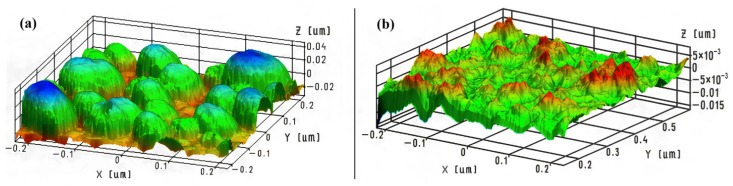
AFM images of silicon surface fragments after its modification with a metal primer: (**a**)—chemical reduction on the silicon surface at 20 °C. Pure blue, green and grey colors corresponds to the 0.02 μm, 0.0 μm and −0.02 μm, correspondingly; (**b**)—chemical reduction on the silicon surface at −20 °C. Pure red and pure green colors corresponds to the 0.005 μm and 0.0 μm, correspondingly.

A polished silicon wafer surface was utilized to evaluate the impact of metal settling temperature on roughness variation. Figure 2 presents comparative AFM pictures of the surface following the deposition of the metal layer at temperatures of +20 °C and −20 °C. A more uniform coating in the height and size of islands was seen at a negative reduction temperature of Ni from a NiSO_4_ solution. Simultaneously, the height of the protrusions diminished from approximately 60 nm (Figure 2a) to 20 nm (Figure 2b). The observed phenomena are clearly linked to a decline in the rate of the nickel reduction chemical reaction as temperature decreases. The likelihood of Ni particles coagulating into bigger clusters due to surface diffusion is diminished.

The electronic structure and phase composition of the initial porous silicon (PSi) layers and samples with precipitated Ni (Ni/PSi) were evaluated via X-ray spectroscopy with an analysis depth of 20–25 nm [15]. Let us turn to the analysis of X-ray emissions of the Si L_2.3_ spectra of the obtained PSi and Ni/PSi samples (Table 1). A comparison of the spectra shows that the main component in the surface layer of the original porous silicon PSi is crystalline silicon, both ordered and disordered (nc-Si, c-Si), as well as silicon oxide (such as SiO_x_ and SiO_2_) and amorphous silicon (a-Si:H).

After Ni deposition, there is a decrease in the phase content of oxidized and amorphous silicon, while the content of crystalline silicon (nc-Si) increases. This may be due to geometric factors such as the size of colloidal particles and the size of metal atoms, as well as the peculiarities of the chemical properties of both the metal atoms themselves and the colloidal solution [15].

### 2.4. Pt Catalyst Synthesis

At the beginning of the development of hydrogen-air fuel cells, platinum black was used, but due to the large size of the particles, catalysts based on it were not efficient enough, and the load of this metal was very high: 5–10 milligrams per square centimeter. To reduce platinum consumption, as well as to increase the efficiency of the catalyst, a solution was proposed related to the deposition of platinum nanoparticles in the region of 2–5 nanometers on carbon [11,16] or metal carriers [2]. A current area of interest is the development of catalysts, where a carrier metal, such as nickel, is located inside the nanoparticle, and the nanoparticle is coated with platinum atoms. Such catalysts are called “core–shell” structures and are highly efficient and can reduce platinum consumption by an order of magnitude.

In order to reduce the temperature–time load in the annealing process necessary for the formation of the catalyst structure, all heat treatments (except for temperatures t ≤ 100 °C) in our experiments were carried out using lamp photon annealing. Photon annealing was carried out on a unit with a maximum irradiation capacity of up to 45 W·cm^−2^. The rate of heating of samples in this case can be (150–200)^0^/s [17]. The required temperature was reached in a few seconds.

**Figure 3:**
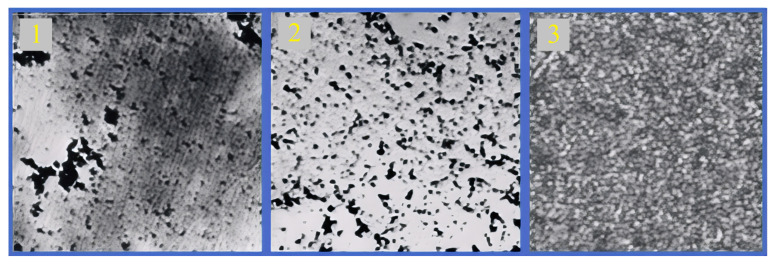
Optical images of the surface of silicon with a platinum catalyst synthesized with different precursors: (**1**) (NH_4_)_2_PtCl_6_ + Isopropanol-2; (**2**) (NH_4_)_2_PtCl_6_ + Ethanol + Ethilenglycol; (**3**) (NH_4_)_2_PtCl_6_ + Formic Acid (the precursors’ exact concentrations are listed in Table 2).

The platinum catalyst on the surface of the pores was formed via thermal reduction from platinum-containing precursors deposited on the inner surface of the Si pores. Table 2 presents the compositions of the precursors used. After impregnation, the electrodes were dried in the air at a temperature of 60–80 °C for 1 h. Subsequent photon annealing made it possible to form the structures depicted in Figure 3 on the silicon surface. The most homogeneous and densely packed structure of the platinum layer was formed on the basis of a precursor with formic acid (number 3 in Table 2).

**Figure 4:**
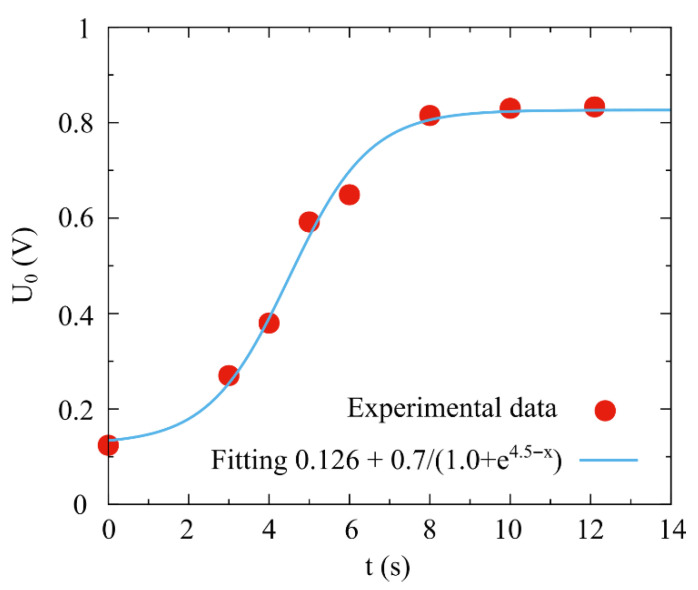
Experimental dependence of U_0_ on the time of photon annealing of the Si electrode with a Pt catalyst on the inner surface of the macropores. The specific radiation power of the lamps is 37 W·cm^−2^.

Figure 4 shows the experimental dependence of U_0_ on the photon annealing time of a porous silicon electrode with a platinum electrocatalyst on the inner surface of the macropores. It can be seen that when annealing a porous silicon electrode, an increase in the value of U_0_ is observed with an increase in time of up to 8 s.

With a further increase in the annealing time, the U_0_ value is practically independent of the etching time. Thus, with the specific power of the lamps of 37 W·cm^−2^ and the annealing time of 8–12 s, the catalytic reaction rate on the cathode electrode is the highest, which is characterized by the highest value of the open circuit voltage (U_0_). Based on these data, this annealing mode was chosen in the experiments.

**Figure 5:**
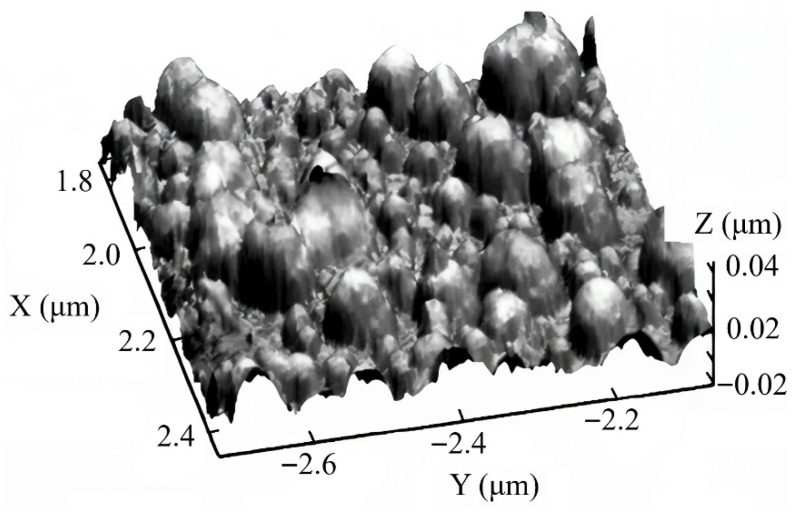
Image of the polished area of the electrode obtained using atomic force microscopy (AFM) after the formation of the “core–shell” structure of the catalyst type Pt@Ni/PSi.

**Figure 6:**
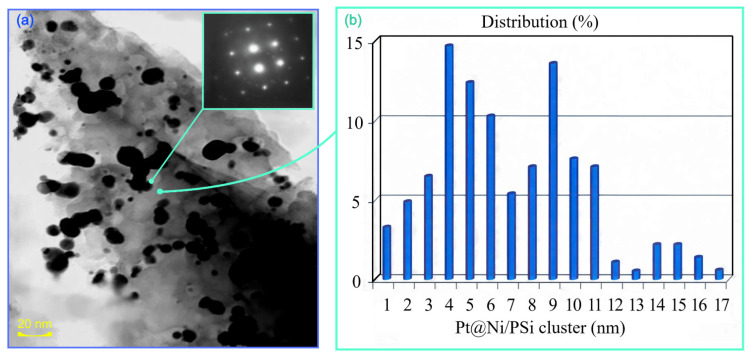
TEM analysis of the core–shell structure of a Pt@Ni/PSi catalyst on the inner surface of the pore wall: (**a**) TEM image of a pore wall with a Pt@Ni/PSi catalyst, the inset shows an electron diffraction pattern from Pt clusters, (**b**) size of core shell clusters, nm.

Figure 5 presents the study results of the structure of the Pt core–shell catalyst, which comprises nanoscale clusters (Figure 6a,b). The electronogram (in the insert of Figure 6a) characterizes the polycrystalline nature of nanoparticles located on the inner surface of the pores of the silicon membrane. The electronogram (in the insert of Figure 6a) characterizes the polycrystalline nature of nanoparticles located on the inner surface of the pores of the silicon membrane.

**Figure 7:**
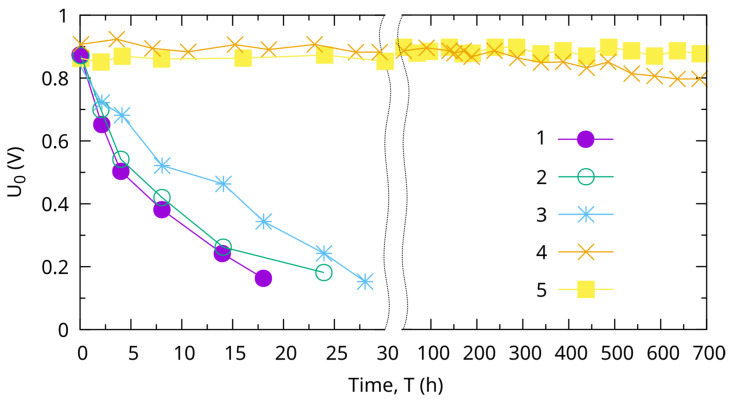
Effectiveness PSi electrodes by catalysts: Pt/PSi (1), Pt/In_2_O_3_ (2), Pt/SnO_2_ (3), Pt/Ni (4) and Pt/Au (5) from the time, determined from the value of the U0 of the “opened electric circuit” half-cell of μ-FC. Dotted vigil line corresponds to time axis breake.

Figure 7 shows the comparative results of the change in the value of the open-circuit voltage U_0_, depending on the operating time of the half-cell of μ-FC with different catalysts. In this paper, the time dependence of efficiency for the structure of μ-FC with a Pt@Ni/PSi catalyst is investigated (Figure 7 number 4). For comparison, Figure 7 shows the experimental dependencies numbered 1, 2, 3, 5 obtained earlier in the paper [2]. The presented results allow us to positively assess the subsequent possibility of practical implementation of the proposed design and technological structure of μ-FC with a composite core–shell Pt catalyst. In a relatively rigorous endurance test mode (tested in a 1.0 M H_2_SO_4_ electrolyte), porous silicon electrodes with a Pt catalyst operated for no more than 20 min (U_0_ decreased from 0.85 V to 0.63 V). In the same mode, electrodes with a Pt@Au/PSi catalyst structure showed stability of U_0_ voltage values for the entire time of measurements (at least 700 h). It should be noted that the technological modes used in the work (temperatures and sedimentation methods, annealing modes, etc.) are more effective for the formation of a Pt@Au/PSi catalyst. Therefore, structures with a Pt@Ni/PSi catalyst showed less variability of the U_0_ value over time. In 700 h, the U_0_ value changed from 0.93 V to 0.82 V. And, although these results characterize a higher durability than those of samples numbered 1, 2, 3, Figure 7. As shown in Figure 7, the Pt@Ni/PSi catalyst formation modes on the porous structure of silicon require further optimization.

## 3. Conclusions

The results of the studies indicate that the main reason for the short-lived functioning of silicon-based μ-FC is the loss of catalytic activity of the Pt catalyst located directly on the silicon structure of the pores. Where the practical implementation of the proposed technical solutions to ensure the necessary durability of the μ-FC based on porous silicon is experimentally demonstrated. The highest stability and durability of operation in the μ-FC structure was also demonstrated by the Pt catalyst with core–shell structure (Pt@Au/Psi).

Replacing gold in the core–shell structure with a Ni sublayer and forming a Pt@Ni/PSi catalyst structure using the technology developed for Pt@Au/PSi shows a higher initial value of open-circuit voltage U_0_ = 0.93 V compared to U_0_ = 0.85 V, which is typical for Pt@Au/PSi. However, after 700 h of operation, this value decreased to 0.83 V. A particularly noticeable decrease was observed after reaching 200–235 h of operation. Such features may indicate the need for additional optimization of the technological modes of the formation of the Pt@Ni/PSi structure.

## Figures and Tables

**Table 1:** Percentage of “phases” in the PSi and PSi/Ni structure. Red and light blue arrows correspond to decreased and increased values correspondingly.

	nc-Si	c-Si	a-Si:H	SiO_x_	SiO_2_	Error, %
PSi	19	5	35	28	13	5
	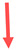	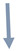	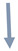	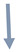	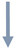	
Ni/PSi	80	0	12	8	0	4

**Table 2:** Composition of platinum-containing precursors.

№	(NH_4_)_2_PtCl_6_	H_2_O	Ethanol	Isopropanol-2	Formic Acid	Ethilenglycol
1	23 mg	0.5 mL	-	3.5 mL	-	-
2	23 mg	0.5 mL	4 mL	-	-	0.15 mL
3	23 mg	0.5 mL	-	-	4 mL	-

## Data Availability

The original contributions presented in this study are included in the article. Further inquiries can be directed to the corresponding author.
